# Involvement of the Penta-EF-Hand Protein Pef1p in the Ca^2+^-Dependent Regulation of COPII Subunit Assembly in *Saccharomyces cerevisiae*

**DOI:** 10.1371/journal.pone.0040765

**Published:** 2012-07-11

**Authors:** Mariko Yoshibori, Tomohiro Yorimitsu, Ken Sato

**Affiliations:** Department of Life Sciences, Graduate School of Arts and Sciences, University of Tokyo, Komaba, Meguro-ku, Tokyo, Japan; Institute of Molecular and Cell Biology, Singapore

## Abstract

Although it is well established that the coat protein complex II (COPII) mediates the transport of proteins and lipids from the endoplasmic reticulum (ER) to the Golgi apparatus, the regulation of the vesicular transport event and the mechanisms that act to counterbalance the vesicle flow between the ER and Golgi are poorly understood. In this study, we present data indicating that the penta-EF-hand Ca^2+^-binding protein Pef1p directly interacts with the COPII coat subunit Sec31p and regulates COPII assembly in *Saccharomyces cerevisiae*. ALG-2, a mammalian homolog of Pef1p, has been shown to interact with Sec31A in a Ca^2+^-dependent manner and to have a role in stabilizing the association of the Sec13/31 complex with the membrane. However, Pef1p displayed reversed Ca^2+^ dependence for Sec13/31p association; only the Ca^2+^-free form of Pef1p bound to the Sec13/31p complex. In addition, the influence on COPII coat assembly also appeared to be reversed; Pef1p binding acted as a kinetic inhibitor to delay Sec13/31p recruitment. Our results provide further evidence for a linkage between Ca^2+^-dependent signaling and ER-to-Golgi trafficking, but its mechanism of action in yeast seems to be different from the mechanism reported for its mammalian homolog ALG-2.

## Introduction

Membrane trafficking is a fundamental mechanism for communication between distinct membrane-bound organelles within a cell. Transport vesicles are used for the delivery of a variety of molecules from one compartment to another. In addition to delivery of protein molecules, these transport vesicles allow exchange of membrane lipids between organelles. Thus, size of the secretory organelle might largely rely on the regulatory mechanisms of vesicular transport that ensure a proper balance of membrane input and output at each organelle. The endoplasmic reticulum (ER)-to-*cis*-Golgi transport step is mediated by coat protein complex II (COPII)-coated vesicles in the anterograde direction, and by COPI-coated vesicles in the retrograde direction [1], [2]. Therefore, the net flux of membrane into and out of the ER, for example, would be determined by the rate of vesicular transport from the *cis*-Golgi, which contributes to input of vesicles, and the rate of vesicular transport out of the ER, which drives vesicle export.

Assembly of the COPII coat is initiated by GDP-GTP exchange on Sar1p catalyzed by the ER-localized transmembrane guanine nucleotide exchange factor (GEF) Sec12p [3]. GTP binding induces a conformational change in Sar1p, which then inserts into the ER membrane [4], [5]. Membrane-bound Sar1p-GTP recruits the Sec23/24p complex by binding to the Sec23p portion, and Sec24p captures the cytoplasmically exposed ER export signal of the transmembrane cargo [6], [7] to form a prebudding complex [8]. The Sec23p subunit is the GTPase-activating protein (GAP) for Sar1p [9], and therefore stimulates Sar1p GTP hydrolysis upon binding to Sar1p, leading to disassembly of the prebudding complex [10]. However, even in the presence of ongoing GTP hydrolysis, binding of Sec23/24p to membranes is stabilized through interactions with transmembrane cargo proteins and continual Sec12p-dependent GTP loading on Sar1p [11], [12]. Subsequently, the outer layer of the COPII coat consisting of Sec13/31p is recruited onto the prebudding complex, which cross-links adjacent prebudding complexes to drive membrane deformation [13], and eventually, COPII vesicles are produced at the specific subdomains of the ER known as ER exit sites (ERES) [14], [15], [16]. The rate of GTP hydrolysis by Sar1p is further accelerated through the binding of Sec13/31p to the prebudding complex, which enhances the Sec23p-mediated GAP activity by an order of magnitude. This activity has been shown to trigger rapid disassembly of the fully assembled coat in a minimal system [10]. Additional factors are thought to regulate the Sar1p-GTPase activity to prevent premature coat disassembly.

While we now know much about the molecular mechanisms of COPII coat assembly and cargo selection, the physiological and mechanistic features of regulation of ER-to-Golgi transport is a relatively unexplored area. Since organelle compartment size and number have been shown to reflect physiological changes, including cell growth, differentiation, and response to stress [17], [18], [19], such compartment homeostasis must be flexible enough to allow rapid and reversible changes. Although signaling and transcriptional events and other higher order processes are certainly involved, it is not surprising that additional factors function through direct regulation of COPII assembly to impact the efficiency of ER export. One such mechanism may be provided by the penta-EF-hand (PEF) Ca^2+^-binding protein apoptosis-linked gene 2 (ALG-2) [20]. Mammalian ALG-2 has been previously implicated in the modulation of COPII-mediated trafficking in a Ca^2+^-dependent manner by binding to the COPII outer shell protein Sec31A at the ERES [21]. Moreover, a recent study showed that ALG-2 plays an inhibitory role in homotypic COPII vesicle fusion by stabilizing Sec31A on the vesicles *in vitro* [22]. These results may provide a link between Ca^2+^ signaling and COPII vesicle biogenesis, but its regulatory mechanisms remain unclear. The *Saccharomyces cerevisiae* genome encodes a single PEF family member, Pef1p, the function of which has not yet been properly defined. Since the fundamental components required for COPII-dependent export from the ER are conserved in yeasts and mammals, the issue arises whether the yeast homolog of ALG-2 (i.e., Pef1p) binds Sec31p and contributes to Ca^2+^-dependent control of COPII-mediated ER-to-Golgi transport in yeast cells. This study reports data demonstrating the direct involvement of Pef1p in the Ca^2+^-dependent regulation of COPII subunit assembly, but in a manner different from that previously observed with its mammalian homolog ALG-2.

## Results

### Ca^2+^-free form of Pef1p binds to the Sec13/31p complex

**Figure 1:**
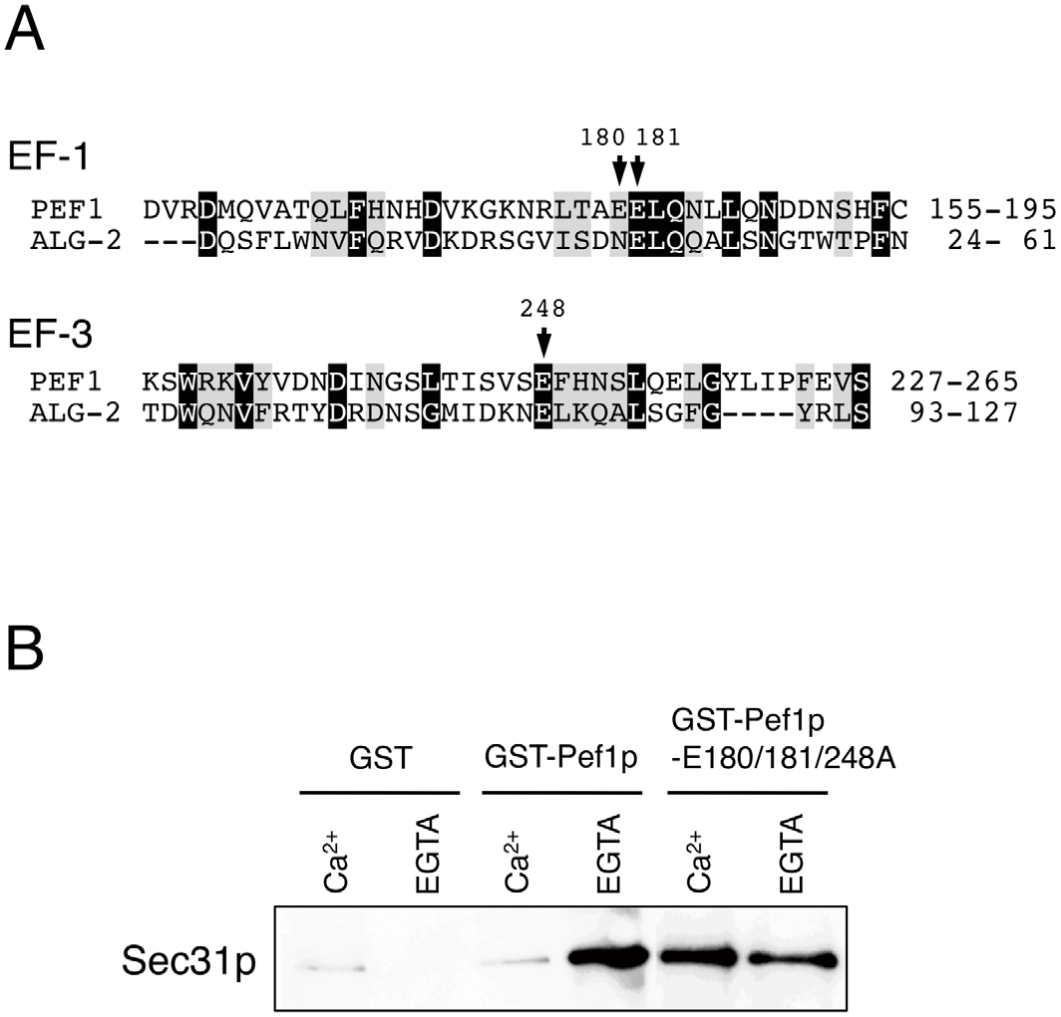
Ca^2+^-free form of Pef1p binds to the Sec13/31p complex. (A) Sequence alignment of the penta-EF-hand motifs of *S. cerevisiae* Pef1p (residues 155–195 and 227–265) and mouse ALG-2 (residues 24–61 and 93–127) obtained by the CLUSTALW program. Highlighted residues (white letters on black) indicate amino acids that are identical to those of ALG-2, and grey residues indicate conservation. Arrows indicate the critical calcium interacting residues E180, E181, and E248. (B) Sec13/31p (80 nM) was incubated with 40 nM of GST, GST-Pef1p, or GST-Pef1p-E180/181/248A in the presence of EGTA (5 mM) or CaCl_2_ (1 mM) as indicated. The reactions were mixed with glutathione-Sepharose beads. After extensive washing, bound proteins were eluted and analyzed by SDS-PAGE and immunoblotting with anti-Sec31p antibody.

To examine the Ca^2+^-regulated binding of Pef1p to Sec31p, we expressed the N-terminally GST-fused Pef1p (GST-Pef1p) in *E. coli*. Affinity-purified GST-Pef1p and control GST immobilized on glutathione-conjugated beads were incubated with purified Sec13/31p in the presence of Ca^2+^ or EGTA. After the beads were washed, the proteins on the beads were immunoblotted with anti-Sec31p antibody. As shown in Fig. 1B, GST-Pef1p indeed pulled down the Sec13/31p complex, whereas GST did not. However, surprisingly, the Ca^2+^ requirement of Pef1p for Sec13/31p binding was found to be completely reversed compared to that observed with mammalian ALG-2 [21]. The binding occurred specifically in the presence of EGTA, indicating that the Ca^2+^-free form of Pef1p preferentially binds to the Sec13/31p.

To further confirm the Ca^2+^ requirement for the interaction of Sec13/31p and Pef1p, we determined the ability of Sec13/31p to bind to a Ca^2+^-binding defective Pef1p mutant. Mammalian ALG-2 loses its Ca^2+^-binding ability as well as its Sec31A-binding ability when critical calcium-interacting glutamic acid residues in the first and third EF-hand motifs were mutated [21], [22]. The corresponding residues are conserved in the EF1 and EF3 motifs of yeast Pef1p (Fig. 1A). We mutated these residues to alanine (Pef1p-E180/181/248A), and the binding capacity of GST-Pef1p-E180/181/248A to Sec13/31p was measured by pull-down assays (Fig. 1B). As expected, the GST-Pef1p-E180/181/248A mutant efficiently pulled down Sec13/31p in the presence of EGTA as well as in the presence of Ca^2+^, thus confirming that the Ca^2+^-free form of Pef1p specifically recognizes and binds to the Sec13/31p complex.

### Sec31p mediates the binding of Pef1p via its proline-rich region

**Figure 2:**
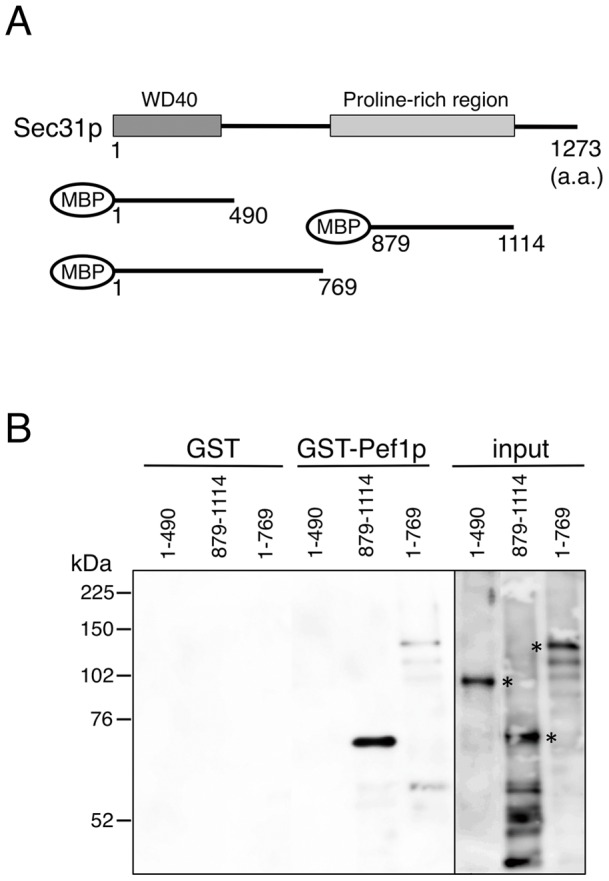
The proline-rich region of the Sec31p subunit is involved in the interaction of the Sec13/31p complex with Pef1p. (A) Schematic representation of the domain structure of Sec31p and the MBP-Sec31p constructs used to identify the region in Sec31p required for Pef1p binding. Numbers indicate amino acid positions defining each deletion construct. (B) The truncated MBP-Sec31p constructs were incubated with GST or GST-Pef1p as indicated. The reactions were mixed with glutathione-Sepharose beads. After extensive washing, bound proteins were eluted and analyzed by SDS-PAGE and immunoblotting with anti-MBP antibody. Asterisks denote the positions of the MBP-Sec31p constructs.

The Sec31p protein can be roughly divided into two distinct domains: the N-terminal WD40 repeat domain essential for binding to Sec13p and the C-terminal proline-rich region required for binding to the Sar1p-Sec23p complex (Fig. 2A) [23]. In a previous study, mammalian ALG-2 has been shown to interact with a proline-rich region of mammalian Sec31A [21]. To map the yeast Sec31p region that is involved in the interaction with Pef1p, we generated deletion mutants of Sec31p as maltose-binding protein (MBP) fusion proteins (Fig. 2A) and investigated their interactions with GST-Pef1p by GST pull-down assays. The three fragments, MBP-Sec31(1–490), MBP-Sec31(1–769), and MBP-Sec31(879–1114) were stably expressed and purified. These fragments were then mixed with glutathione beads coated with either GST or GST-Pef1p. After extensive washing, the proteins retained on the beads were subjected to immunoblotting with anti-MBP antibodies. As shown in Fig. 2B, only the MBP-Sec31(879–1114) fragment, which corresponds to the proline-rich region, was able to interact with Pef1p. These data demonstrate that the proline-rich region of the Sec31p subunit plays a key role in the interaction of the yeast Sec13/31p complex with Pef1p, as has been observed with mammalian ALG-2.

### Pef1p slows down Sec13/31p recruitment to the membrane

**Figure 3:**
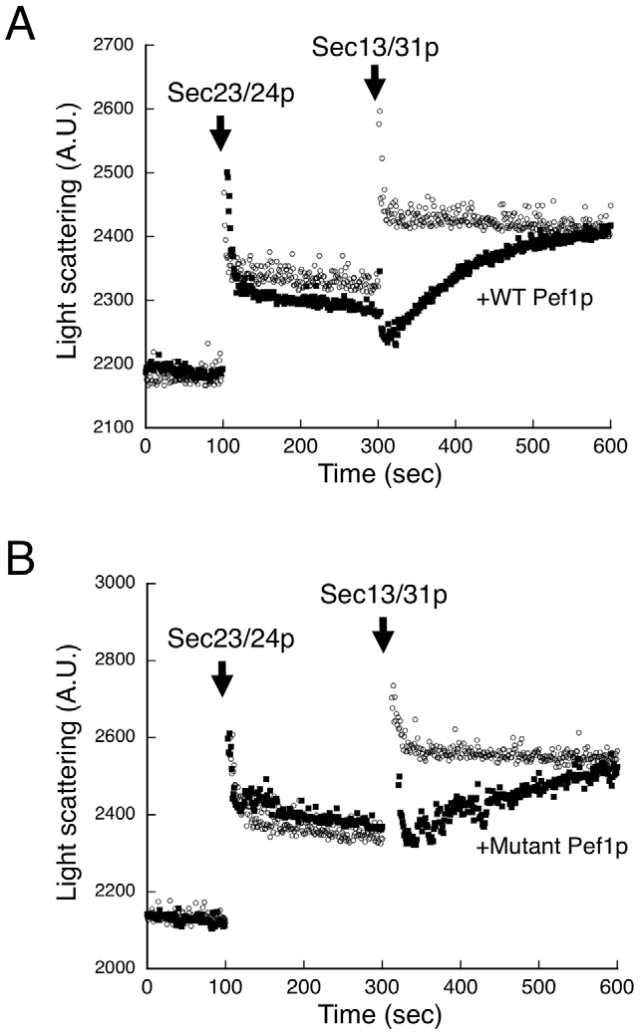
Assembly of the COPII coat onto liposomes in the presence of Pef1p. The reaction initially contained liposomes (100 µg/mL), Sar1p (950 nM), and GMP-PNP (0.1 mM) in the presence (closed circles) or absence (open circles) of GST-Pef1p (520 nM) (A) or GST-Pef1p-E180/180/248A (520 nM) (B). EGTA (5 mM) was included in (A). After preincubation, Sec23/24p (160 nM) and Sec13/31p (260 nM) were added at the indicated time points, and the light scattering of the suspension was monitored.

To directly determine the functional consequences of the Pef1p-Sec13/31p interaction, we next tested whether the presence of Pef1p affects the recruitment of Sec13/31p to the membrane. Because the proline-rich region of Sec31 is involved in the interaction with the Sar1/Sec23 complex [24], it is possible that the association of Pef1p with this region hinders Sec13/31p recruitment to the membrane. The real-time assembly of individual COPII coat subunits on chemically defined liposomes can be monitored and analyzed by light scattering [10]. Binding and dissociation of the COPII coat components onto liposomes lead to changes in mass and are detectable by scattered light intensity. The sequential addition of Sec23/24p and Sec13/31p to liposomes preloaded with Sar1p-GMP-PNP induced an instantaneous increase in the light scattering signal, which corresponds to the rapid recruitment of each COPII component as previously reported (Fig. 3). Addition of Sec23/24p in the presence of Pef1p with EGTA also yielded a rapid increase in the light scattering signal that was almost identical to that observed in the absence of Pef1p, indicating that Pef1p has no significant effect on Sec23/24p recruitment. In contrast, the rate of Sec13/31p recruitment to Sec23/24p-loaded liposomes was significantly reduced when an equimolar amount of Pef1p to Sec13/31p was present with EGTA (Fig. 3A). Higher molar ratios of Pef1p did not further influence the rate of Sec13/31p binding (data not shown). It should be noted that the final plateau yield of the binding signal was similar to that obtained in the absence of Pef1p. These results suggest that the Ca^2+^-free form of Pef1p binds to the proline-rich region of the Sec31p subunit in the Sec13/31p complex and slows down, but does not completely abrogate, Sec13/31p recruitment to the membrane. Similar results were obtained with the Ca^2+^-binding deficient mutant Pef1p-E180/181/248A in the absence of EGTA (Fig. 3B), confirming the Ca^2+^ specificity of this response.

**Figure 4:**
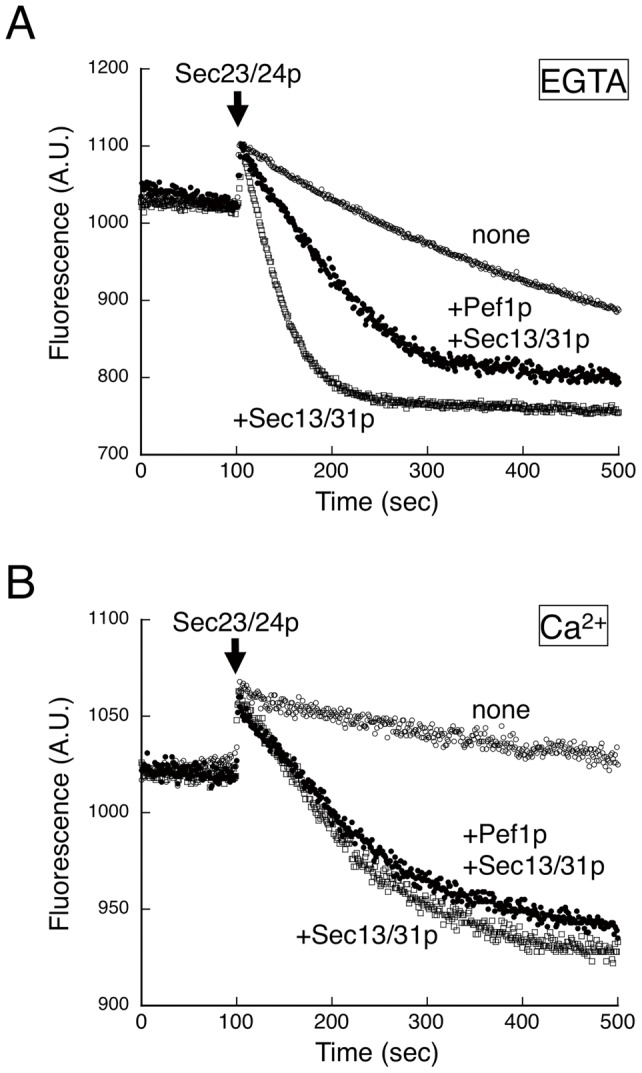
GAP stimulation activity of Sec31p in the presence of Pef1p. The reaction initially contained liposomes (50 µg/mL), Sar1p (500 nM), GTP (0.1 mM), and either EGTA (5 mM) (A) or CaCl_2_ (1 mM) (B) in the presence of Sec13/31p (50 nM) (open squares) or both Pef1p (70 nM) and Sec13/31p (50 nM) (closed circles). After preincubation, Sec23/24p (160 nM) was added at the indicated time point. Transition of Sar1p from the GTP-bound to the GDP-bound state was monitored by tryptophan fluorescence of Sar1p at 340 nm.

To further evaluate the influence of Pef1p binding to the Sec13/31p complex, we investigated whether the presence of Pef1p affects Sec31p-mediated stimulation of the GAP activity of Sec23p during COPII assembly. We tested Sar1p GTPase activity in the presence of Pef1p by using a real-time tryptophan fluorescence assay that monitors the GDP/GTP state of Sar1p (Fig. 4). As expected, Pef1p with EGTA markedly diminished, but did not completely abolish the Sec13/31p-stimulated GTPase activity of Sar1p (Fig. 4A), although the presence of Ca^2+^ did not significantly influence the activity (Fig. 4B). These results correlate well with the data from the light scattering measurements (Fig. 3), which showed that Pef1p binding does not completely block Sec13/31p recruitment but serves to significantly slow the process.

### Overexpression of Pef1p causes a growth defect, which is not due to anterograde transport inhibition

**Figure 5:**
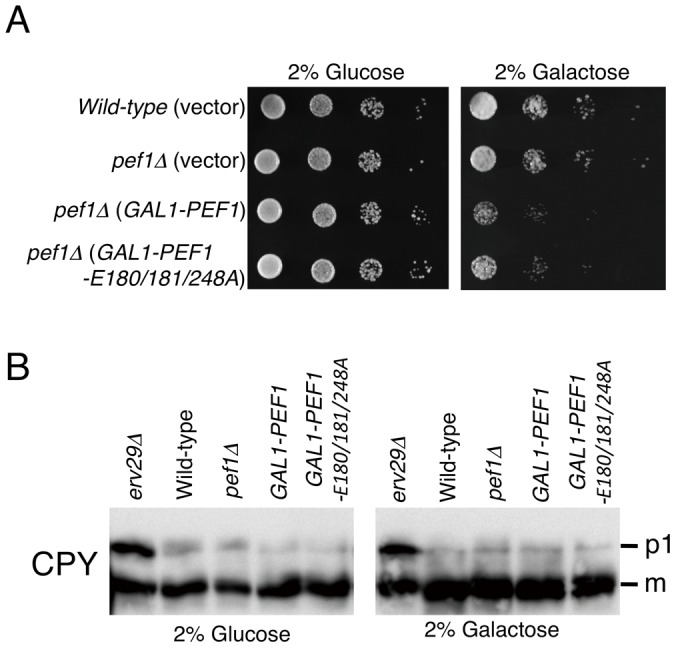
Pef1p overexpression causes defects in yeast cell growth, but does not affect anterograde transport. (A) Isogenic wild-type (BY4741) or *pef1*Δ cells transformed with *GAL1-PEF1* constructs or vector (pYES2) as indicated were grown to saturation at 30°C, adjusted to an OD_600_ of 0.5, and 5 µL of a 10-fold dilution series was spotted onto selective plates containing 2% glucose (left panel) or 2% galactose (right panel). (B) Wild-type and *pef1*Δ cells transformed with the same plasmids as in (A) were grown at 30°C to log phase in selective media supplemented with 2% glucose (left panel) or 2% galactose (right panel) to induce overexpression of the indicated Pef1p forms. Equal amounts of whole cell extracts were subjected to immunoblotting with anti-CPY antibody. The *erv29*Δ strain was used as a control. The ER (p1) and mature (m) forms of CPY are indicated.

To explore the function of Pef1p in regulating anterograde transport *in vivo*, we placed *PEF1* or *PEF1-E180/181/248A* under control of the inducible *GAL1* promoter and determined the effect of overexpression. A strain carrying a chromosomal deletion of *PEF1* was previously found to have no growth defect [25]. Consistent with these data, *pef1*Δ cells exhibited a growth rate comparable to that of the isogenic wild-type strain (Fig. 5A, left panel). In the case of Pef1p or Pef1p-E180/181/248A overexpression, we observed that increases in galactose concentration significantly reduced growth rates compared with the wild-type cells (Fig. 5A, right panel). To determine if the cause of this toxicity is related to defective intracellular transport, we monitored transport of the vacuolar protease carboxypeptidase Y (CPY) as a marker for early events in the secretory pathway. As shown in Fig. 5B, overexpression of Pef1p or Pef1p-E180/181/248A did not show accumulation of the ER form of CPY (p1 form) indistinguishable from that of wild-type cells. For comparison, the delayed trafficking of CPY in cells lacking *ERV29*, which encodes a sorting receptor for CPY export from the ER [26], showed the accumulation of the p1 form of CPY. These findings suggest that the overexpression of Pef1p or Pef1p-E180/181/248A does not block anterograde ER-to-Golgi trafficking, and thus, the growth defect induced by overexpression of Pef1p or Pef1p-E180/181/248A is not simply due to ineffective transport from the ER to the Golgi apparatus.

### Cellular localization of Pef1p in S. cerevisiae cells

**Figure 6:**
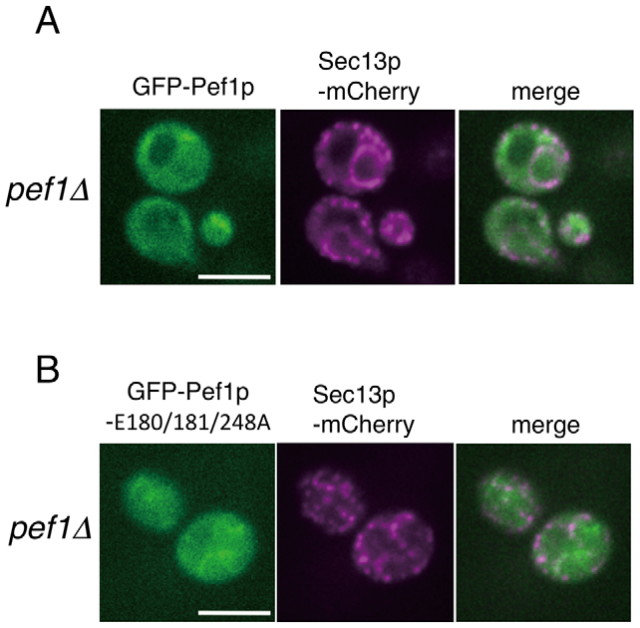
Cellular localization of Pef1p. Log-phase cultures of *pef1*Δ mutant cells co-expressing Sec13p-mCherry and either GFP-Pef1p (A) or GFP-Pef1p-E180/181/248A (B) were visualized by confocal microscopy. The panels on the right show merged fluorescence images. Scale bars, 5 µm.

Mammalian ALG-2 has been reported to be localized both in the cytosol and at the ERES via a calcium-dependent association with Sec31A [21]. To assess the cellular localization of Pef1p in yeast cells, we expressed GFP-tagged Pef1p in *pef1*Δ cells under the control of its own promoter on a single-copy plasmid. Plasmid-borne GFP-Pef1p complemented the SDS sensitivity of the *pef1*Δ strain [25], suggesting that this tagged version is functional (data not shown). We examined the localization of GFP-Pef1p by co-labeling with its binding partner Sec13p-mCherry, which also marks the ERES [27], [28], [29]. GFP-Pef1p was diffusely localized throughout the cytosol and did not overlap with the Sec13p-mCherry signal (Fig. 6A). We also observed the localization of the GFP-Pef1p-E180/181/248A mutant in *pef1*Δ cells; the majority of the GFP signal was found in the cytosol, but was not significantly concentrated at the ERES (Fig. 6B). Together, these results indicate that the ER exit site is not the major localization site of Pef1p in yeast cells.

## Discussion

Our results indeed illustrate that the yeast homolog of ALG-2, i.e., Pef1p, also binds to Sec31p and modulates COPII coat assembly. Mammalian ALG-2 has also been shown to interact with proline-rich regions of Alix/AIP1 [30], [31], annexins VII and XI [32], and tumor susceptibility gene 101 [33] in a Ca^2+^-dependent manner. We demonstrated that Pef1p specifically binds to the proline-rich region of Sec31p (Fig. 2). Thus, the mechanism of the interaction between Pef1p and Sec31p would be similar to that of other PEF proteins. Nevertheless, the Ca^2+^ dependency for the Sec31p interaction with Pef1p appears to be reversed compared to that previously reported for the ALG-2/Sec31A interaction. The results of our pull-down experiments clearly demonstrated the preferential affinity of Sec31p for Pef1p in the absence of Ca^2+^ (Fig. 1), while ALG-2 has been shown to bind Sec31A in a calcium-dependent manner. However, the effect on COPII also seems to be reversed: ALG-2 binding stabilizes the membrane association of Sec31A, whereas Pef1p binding prevents the membrane recruitment of Sec13/31p. Although sequence alignment of the C-terminal calcium binding domains of Pef1p shows a high conservation with that of ALG-2, Pef1p has a relatively long N-terminal extension [25], which might potentially contribute to the difference in their behavior. From these data, we postulated that the differing Ca^2+^ dependency could be explained by a different ER-to-Golgi trafficking route between yeasts and mammals.

In mammalian cells, COPII vesicles that bud from the ER do not directly fuse to the Golgi apparatus; instead, they are thought to tether to each other to form pre-Golgi intermediates, also termed vesicular tubular clusters (VTCs) or ER-Golgi intermediate compartment (ERGIC). The lumen of the ER and Golgi contain a high concentration of free Ca^2+^ due to Ca^2+^-ATPase pump activity [34], [35], and the cytoplasmic Ca^2+^ concentration immediately adjacent to the membranes of these organelles is reported to be relatively high, possibly because of nonspecific basal leakage of luminal Ca^2+^ [36], [37]. However, since Ca^2+^-ATPases are not incorporated into COPII vesicles, pre-Golgi intermediates exhibit lower Ca^2+^ signatures [38]. With these features, ALG-2 may stabilize the Sec13/31p coat at ER exit sites via a Ca^2+^-dependent interaction to promote COPII coat assembly, while simultaneously encouraging coat disassembly before vesicle fusion with the Ca^2+^-poor pre-Golgi intermediates.

In contrast, COPII vesicles in yeasts are thought to tether and directly fuse with the *cis*-Golgi. From this picture, if Pef1p in *S. cerevisiae* had the same Ca^2+^ dependence as ALG-2 and behaved as it does in mammalian cells, then Pef1p would inhibit the dissociation of the outer Sec13/31p coat from COPII vesicles at the surface of *cis*-Golgi membranes. However, our *in vitro* binding experiments showed that Pef1p requires low calcium levels for Sec31p binding, and thus, Pef1p in yeast may not act at the membrane surface of these Ca^2+^-rich organelles. Instead, the low concentration of calcium in the cytosol area could promote Pef1p binding to Sec13/31p. Consistent with this hypothesis, we found that GFP-Pef1p exclusively localizes to the cytosol (Fig. 6). Recent work suggests that the inner COPII coat, i.e., the Sec23/24p complex, directly participates in tethering COPII vesicles to the Golgi membranes by binding directly to a component of the tethering complex TRAPP, and therefore, it would be released only after delivery of vesicles to the Golgi compartment [39]. In contrast to the inner layer coat, disassembly of the outer COPII coat Sec13/31p does not necessarily occur after vesicle tethering. Therefore, it is possible that Pef1p binding to Sec13/31p in the cytosol would tend to discourage back-recruitment of Sec13/31p that had been released from the COPII vesicles, although the timing of Sec13/31p disassembly and *in vivo* triggers for uncoating still remain unknown. Given these assumptions, perhaps the failure to exhibit severe ER-to-Golgi transport defects in the presence of excess Pef1p is not surprising (see below).

We observed that overexpression of either wild-type Pef1p or the Pef1p-E180/181/248A mutant from the *GAL1* promoter resulted in growth inhibition, but had no effect on ER-to-Golgi transport (Fig. 5). These findings suggest that excessive levels of Pef1p may influence steps other than ER-to-Golgi transport. Penta-EF-hand proteins have been shown to participate in a variety of calcium-dependent processes in vertebrates [20]. Pef1p is the only PEF family protein found in the budding yeast *S. cerevisiae*, and is likely to play multiple roles in the control of cellular physiology, such as polar bud growth and cell wall abscission [25]. Therefore, we reasoned that the mechanism of the overexpression phenotype of Pef1p might be that the proper balance of components required for these cellular processes was changed, which limits our ability to further examine the contribution of Pef1p to intracellular transport *in vivo*. Although we have shown here that the Ca^2+^-free Pef1p-E180/181/248A mutant constitutively binds to the Sec13/31p complex, overexpression of this mutant did not prevent *in vivo* ER-to-Golgi transport. This result is quite plausible because our light-scattering assays demonstrated that the Pef1p-E180/181/248A mutant delays, rather than blocks, the recruitment of Sec13/31p (Fig. 3), and this mutant is likely to act as a kinetic inhibitor in this reaction. These data are consistent with our results that, unlike ALG-2, GFP-Pef1p-E180/181/248A did not colocalize with Sec13p-mCherry at the ERES (Fig. 6).

Definition of the precise function of Pef1p in yeast cells will clearly require further experimentation, but Pef1p is the first coat-binding protein that negatively regulates coat assembly. The kinetic inhibition of Sec13/31p recruitment may represent a novel regulatory mechanism controlling the rate of ER-to-Golgi transport. Comprehending these refinements and additional levels of control is crucial to our understanding of the molecular basis of ER export in the context of the entire secretory pathway.

## Materials and Methods

### Yeast strains and plasmids

*S. cerevisiae* strains used in this study were BY4741 (*MATa his3*Δ*1 leu2*Δ*0 met15*Δ*0 ura3*Δ*0*) and BY4741 *pef1::KanMX6* (Research Genetics). Unless otherwise noted, cultures were grown at 30°C in YPD medium (1% Bacto-yeast extract (Difco Laboratories, Inc.), 2% polypeptone (Nihon Seiyaku), and 2% dextrose), MVD medium (0.67% yeast nitrogen base without amino acids (Difco Laboratories, Inc.), and 2% dextrose), or MCD medium, which is MVD containing 0.5% casamino acids, supplemented appropriately. The coding sequence of Pef1p was generated by PCR from *S. cerevisiae* genomic DNA and inserted into the *Sal*I-*Not*I sites of the *Escherichia coli* expression vector pGEX-4T-1 (GE Healthcare), yielding pMY3 (GST-Pef1p). The triple mutations to alanine at positions 180/181/248 (E180/181/248A) were introduced into the gene of GST-Pef1p in plasmid pMY3 by using primer-directed mutagenesis, yielding pMY14. The coding sequences of truncated Sec31p (residues 1–490, residues 879–1114, or residues 1–769) were amplified by PCR from genomic DNA and inserted into the *Bam*HI-*Hind*III sites of the *E. coli* expression vector pMAL-c2x (New England BioLabs), yielding pMY11 (MBP-Sec31(1–490)), pMY12 (MBP-Sec31(879–1114)), and pMY13 (MBP-Sec31(1–769)), respectively. The coding sequence of the *PEF1* gene, together with 400 bp upstream and downstream, was amplified from genomic DNA by PCR and inserted into the *Sal*I site of pRS316, yielding pMYy10. The E180/181/248A mutations were introduced into the *PEF1* gene in the pMYy10 plasmid by using primer-directed mutagenesis, yielding pMYy13. A *Sph*I site was created just before the start codon in pMYy10 and pMYy13, and the fragment encoding AcGFP, which was PCR-amplified from pAcGFP1 (Clonetech), was inserted into these *Sph*I sites to yield pMYy13 (GFP-Pef1p) and pMYy14 (GFP-Pef1p-E180/181/248A). The coding sequences of *PEF1* and *PEF1-E180/181/248A* were amplified by PCR from pMYy10 and pMYy13, respectively, and inserted into the *Hind*III-*Not*I sites of pYES2 (Invitrogen) downstream of the *GAL1* promoter, yielding pMYy19 and pMYy20, respectively. The coding sequence of the *SEC13* gene, together with upstream and downstream sequences, was amplified from genomic DNA by PCR and inserted into the *Sac*I-*Xho*I sites of pRS315. A *Bam*HI site was created just before the stop codon, and the fragment encoding mCherry, which was PCR-amplified from pmCherry (Clontech), was inserted into this *Bam*HI site to yield pSEC13-5G-mCherry.

### Protein expression and purification

Sar1p, Sec23/24p, and Sec13/31p were prepared as previously described [11]. GST-Pef1p and GST-Pef1p-E180/181/248A were purified from *E. coli* JM109 cells carrying the pMY3 and pMY14 plasmids, respectively. The cells were cultured at 25°C and induced with isopropyl-1-thio-ß-D-glucopyranoside (IPTG; 1 mM) overnight. The cells were collected and washed with buffer A (20 mM HEPES-KOH, pH 8.0, 160 mM potassium acetate). The total cell lysate was prepared by sonication in lysis buffer (20 mM HEPES-KOH, pH 8.0, 160 mM potassium acetate, 0.2% (w/v) Triton X-100, 1 mM MgCl_2_) supplemented with a protease inhibitor cocktail (Roche Complete, EDTA-free). After removal of insoluble materials by centrifugation, the supernatant was incubated with glutathione-Sepharose beads (GE Healthcare) at 4°C for 1 h. The beads were washed with buffer A, and then GST-Pef1p or GST-Pef1p-E180/181/248A were eluted with 10 mM glutathione in buffer A. MBP-fused truncated Sec31p proteins were purified from *E. coli* C41(DE3) cells carrying the pMY11, pMY12, or pMY13 plasmids. After a 4-h induction with IPTG (1 mM) at 30°C, the total cell lysate was prepared using the above method, except that buffer B (20 mM HEPES-KOH, pH 8.0, 160 mM potassium acetate, 5 mM EDTA) was used instead of buffer A. The cleared lysate was mixed with amylose resin (New England BioLabs), washed with buffer B, and then MBP-Sec31p fragments were eluted with buffer A containing 30 mM maltose.

### Preparation of liposomes

Major-minor mix liposomes [40] were prepared as previously described [41]. Briefly, phospholipids were mixed with octylglucoside, and liposomes were formed by detergent dialysis, followed by flotation in a Nycodenz density gradient.

### Pull-down assays

GST-Pef1p or GST-Pef1p-E180/181/248A (40 nM) was mixed with Sec13/31p (80 nM) or MBP-Sec31p fragment (80 nM) in 400 µL of binding buffer C (20 mM HEPES-KOH, pH 8.0, 160 mM potassium acetate, 0.1% (w/v) Triton X-100) in the presence of either CaCl_2_ (1 mM) or EGTA (5 mM) and incubated with glutathione-Sepharose beads at 4°C for 2 h. The beads were washed three times with the same buffer, and the bound material was eluted with 10 mM glutathione in buffer C in the presence of either CaCl_2_ (1 mM) or EGTA (5 mM). Samples were analyzed by SDS-PAGE and immunoblotting.

### Tryptophan fluorescence and light scattering measurements

Tryptophan fluorescence (excitation, 298 nm; emission, 340 nm) and light scattering (λ = 350 nm) were measured in a quartz cuvette (total volume, 200 µL) with a Hitachi fluorescence spectrophotometer (F-2500) equipped with a thermostatically controlled cell holder and magnetic stirrer [42]. Reactions were performed in buffer containing 20 mM HEPES-KOH, pH 6.8, 160 mM potassium acetate, and 1 mM MgCl_2_. All experiments were performed at 25°C.

### Microscopy

Yeast cells expressing AcGFP- or mCherry-fused proteins were grown to the mid-log phase. Fluorescence microscopy observation was carried out using an Olympus IX71 microscope equipped with a CSU10 spinning-disk confocal scanner (Yokogawa Electric Corporation) and an electron multiplying charge coupled device camera (iXon, DU897, Andor Technology). The acquired images were analyzed by Andor iQ (Andor Technology). In this setting, a 473 nm solid-state laser (Showa Optronics, J050BS) was used to excite AcGFP and mCherry at 561 nm (Jive, Cobolt).

## References

[1] Dancourt J, Barlowe C (2010). Protein sorting receptors in the early secretory pathway.. Annu Rev Biochem.

[2] Zanetti G, Pahuja KB, Studer S, Shim S, Schekman R (2012). COPII and the regulation of protein sorting in mammals.. Nat Cell Biol.

[3] Barlowe C, Schekman R (1993). *SEC12* encodes a guanine-nucleotide-exchange factor essential for transport vesicle budding from the ER.. Nature.

[4] Huang M, Weissman JT, Beraud-Dufour S, Luan P, Wang C (2001). Crystal structure of Sar1-GDP at 1.7 A resolution and the role of the NH2 terminus in ER export.. J Cell Biol.

[5] Bi X, Corpina RA, Goldberg J (2002). Structure of the Sec23/24-Sar1 pre-budding complex of the COPII vesicle coat.. Nature.

[6] Mossessova E, Bickford LC, Goldberg J (2003). SNARE selectivity of the COPII coat.. Cell.

[7] Miller EA, Beilharz TH, Malkus PN, Lee MC, Hamamoto S (2003). Multiple cargo binding sites on the COPII subunit Sec24p ensure capture of diverse membrane proteins into transport vesicles.. Cell.

[8] Kuehn MJ, Herrmann JM, Schekman R (1998). COPII-cargo interactions direct protein sorting into ER-derived transport vesicles.. Nature.

[9] Yoshihisa T, Barlowe C, Schekman R (1993). Requirement for a GTPase-activating protein in vesicle budding from the endoplasmic reticulum.. Science.

[10] Antonny B, Madden D, Hamamoto S, Orci L, Schekman R (2001). Dynamics of the COPII coat with GTP and stable analogues.. Nat Cell Biol.

[11] Sato K, Nakano A (2005). Dissection of COPII subunit-cargo assembly and disassembly kinetics during Sar1p-GTP hydrolysis.. Nat Struct Mol Biol.

[12] Futai E, Hamamoto S, Orci L, Schekman R (2004). GTP/GDP exchange by Sec12p enables COPII vesicle bud formation on synthetic liposomes.. EMBO J.

[13] Tabata KV, Sato K, Ide T, Nishizaka T, Nakano A (2009). Visualization of cargo concentration by COPII minimal machinery in a planar lipid membrane.. EMBO J.

[14] Orci L, Ravazzola M, Meda P, Holcomb C, Moore HP (1991). Mammalian Sec23p homologue is restricted to the endoplasmic reticulum transitional cytoplasm.. Proc Natl Acad Sci USA.

[15] Bannykh SI, Rowe T, Balch WE (1996). The organization of endoplasmic reticulum export complexes.. J Cell Biol.

[16] Yorimitsu T, Sato K (2012). Insights into structural and regulatory roles of Sec16 in COPII vesicle formation at ER exit sites.. Mol Biol Cell 23: in press.

[17] Lee TH, Linstedt AD (1999). Osmotically induced cell volume changes alter anterograde and retrograde transport, Golgi structure, and COPI dissociation.. Mol Biol Cell.

[18] Lu Z, Joseph D, Bugnard E, Zaal KJ, Ralston E (2001). Golgi complex reorganization during muscle differentiation: visualization in living cells and mechanism.. Mol Biol Cell.

[19] Colanzi A, Suetterlin C, Malhotra V (2003). Cell-cycle-specific Golgi fragmentation: how and why?. Curr Opin Cell Biol.

[20] Maki M, Kitaura Y, Satoh H, Ohkouchi S, Shibata H (2002). Structures, functions and molecular evolution of the penta-EF-hand Ca2+-binding proteins.. Biochim Biophys Acta.

[21] Yamasaki A, Tani K, Yamamoto A, Kitamura N, Komada M (2006). The Ca2+-binding protein ALG-2 is recruited to endoplasmic reticulum exit sites by Sec31A and stabilizes the localization of Sec31A.. Mol Biol Cell.

[22] Bentley M, Nycz DC, Joglekar A, Fertschai I, Malli R (2010). Vesicular calcium regulates coat retention, fusogenicity, and size of pre-Golgi intermediates.. Mol Biol Cell.

[23] Fath S, Mancias JD, Bi X, Goldberg J (2007). Structure and organization of coat proteins in the COPII cage.. Cell.

[24] Bi X, Mancias JD, Goldberg J (2007). Insights into COPII coat nucleation from the structure of Sec23.Sar1 complexed with the active fragment of Sec31.. Dev Cell.

[25] Vernarecci S, Colotti G, Ornaghi P, Schiebel E, Chiancone E (2007). The yeast penta-EF protein Pef1p is involved in cation-dependent budding and cell polarization.. Mol Microbiol.

[26] Belden WJ, Barlowe C (2001). Role of Erv29p in Collecting Soluble Secretory Proteins into ER-Derived Transport Vesicles.. Science.

[27] Castillon GA, Watanabe R, Taylor M, Schwabe TM, Riezman H (2009). Concentration of GPI-anchored proteins upon ER exit in yeast.. Traffic.

[28] Shindiapina P, Barlowe C (2010). Requirements for transitional endoplasmic reticulum site structure and function in Saccharomyces cerevisiae.. Mol Biol Cell.

[29] Okamoto M, Kurokawa K, Matsuura-Tokita K, Saito C, Hirata R (2012). High-curvature domains of the ER are important for the organization of ER exit sites in Saccharomyces cerevisiae.. J Cell Sci: in press.

[30] Missotten M, Nichols A, Rieger K, Sadoul R (1999). Alix, a novel mouse protein undergoing calcium-dependent interaction with the apoptosis-linked-gene 2 (ALG-2) protein.. Cell Death Differ.

[31] Vito P, Pellegrini L, Guiet C, D'Adamio L (1999). Cloning of AIP1, a novel protein that associates with the apoptosis-linked gene ALG-2 in a Ca2+-dependent reaction.. J Biol Chem.

[32] Satoh H, Nakano Y, Shibata H, Maki M (2002). The penta-EF-hand domain of ALG-2 interacts with amino-terminal domains of both annexin VII and annexin XI in a Ca2+-dependent manner.. Biochim Biophys Acta.

[33] Katoh K, Suzuki H, Terasawa Y, Mizuno T, Yasuda J (2005). The penta-EF-hand protein ALG-2 interacts directly with the ESCRT-I component TSG101, and Ca2+-dependently co-localizes to aberrant endosomes with dominant-negative AAA ATPase SKD1/Vps4B.. Biochem J.

[34] Pinton P, Pozzan T, Rizzuto R (1998). The Golgi apparatus is an inositol 1,4,5-trisphosphate-sensitive Ca2+ store, with functional properties distinct from those of the endoplasmic reticulum.. EMBO J.

[35] Petersen OH, Tepikin A, Park MK (2001). The endoplasmic reticulum: one continuous or several separate Ca(2+) stores?. Trends Neurosci.

[36] Wahl M, Sleight RG, Gruenstein E (1992). Association of cytoplasmic free Ca2+ gradients with subcellular organelles.. J Cell Physiol.

[37] Camello C, Lomax R, Petersen OH, Tepikin AV (2002). Calcium leak from intracellular stores–the enigma of calcium signalling.. Cell Calcium.

[38] Pezzati R, Bossi M, Podini P, Meldolesi J, Grohovaz F (1997). High-resolution calcium mapping of the endoplasmic reticulum-Golgi-exocytic membrane system. Electron energy loss imaging analysis of quick frozen-freeze dried PC12 cells.. Mol Biol Cell.

[39] Cai H, Yu S, Menon S, Cai Y, Lazarova D (2007). TRAPPI tethers COPII vesicles by binding the coat subunit Sec23.. Nature.

[40] Matsuoka K, Orci L, Amherdt M, Bednarek SY, Hamamoto S (1998). COPII-coated vesicle formation reconstituted with purified coat proteins and chemically defined liposomes.. Cell.

[41] Sato K, Nakano A (2004). Reconstitution of coat protein complex II (COPII) vesicle formation from cargo-reconstituted proteoliposomes reveals the potential role of GTP hydrolysis by Sar1p in protein sorting.. J Biol Chem.

[42] Kodera C, Yorimitsu T, Nakano A, Sato K (2011). Sed4p stimulates Sar1p GTP hydrolysis and promotes limited coat disassembly.. Traffic.

